# Preventing suicide among older women in China: the integration of gamified group-based exercise in a virtual reality environment

**DOI:** 10.3389/fpsyg.2025.1626476

**Published:** 2025-09-08

**Authors:** Jinjin Zhang

**Affiliations:** Zhengzhou Technology and Business University, Zhengzhou, China

**Keywords:** mental health, gamified exercise, social exercise intervention, VR training, elderly women

## Abstract

**Objective:**

Suicide among elderly women has increasingly been recognized as a significant public health concern, necessitating the development of innovative, evidence-based interventions tailored to this vulnerable population. This study aimed to examine the effectiveness of gamified group-based physical activity in a virtual reality (VR) environment for enhancing psychological resilience and reducing suicidal ideation among elderly women.

**Methods:**

A randomized controlled trial (RCT) design with convenience sampling was adopted for this study. A total of 120 elderly women with clinically confirmed suicidal ideation were recruited through purposive announcements at senior care facilities in Beijing, China, and were then randomly allocated into experimental (*n* = 60) and control (*n* = 60) groups using a computer-generated randomization schedule. Data were collected using two validated questionnaires: the Subjective Vitality Scale and the Life Hope Questionnaire. The normality of data distribution was assessed using the Kolmogorov–Smirnov test, and the homogeneity of variances across groups was evaluated via Levene’s test. To control for baseline differences and assess the impact of the intervention, data were analyzed using analysis of covariance.

**Results:**

The findings revealed statistically significant improvements in both subjective vitality and life hope scores among participants in the experimental group compared to those in the control group (*p* < 0.01). The effect size for hopefulness was η^2^ = 0.29, and the effect size for mental vitality was η^2^ = 0.21.

**Conclusion:**

These findings highlight that gamified VR-based group exercise is a promising non-pharmacological approach for improving psychological well-being and mitigating suicide risk in elderly women. By combining immersive technology, social engagement, and motivational components, such interventions offer an effective, engaging, and scalable strategy to address mental health challenges in aging populations.

## 1 Introduction

Suicide among the elderly is recognized as a major public health challenge worldwide. In China, this issue has become particularly prominent due to the country’s large aging population. Estimates indicate that the elderly population in China (aged 65 and above) has the highest suicide rate among all age groups, with rates reported to be four to five times higher than those of the general population (Dong et al., 2015). In recent years, this trend has been particularly alarming in rural areas; for example, in 2021, the suicide rate among rural elderly individuals aged 65 and above was reported to range between 14 and 39 per 100,000 people, significantly exceeding that of younger age groups (Zhang et al., 2024). Notably, unlike many Western countries where late-life suicide predominantly occurs among men, elderly women in China are also severely affected. Reports suggest that the suicide rate among elderly women is approximately 25% higher than that of their male counterparts (Wu et al., 2024). One of the major psychological factors associated with suicidal behavior in the elderly is hopelessness, and studies have shown that older adults living in rural areas with high levels of hopelessness are over seven times more likely to engage in suicidal behavior compared to their peers with lower levels of hopelessness (Stødle et al., 2019). Moreover, social changes such as the restructuring of family dynamics and the migration of younger generations to distant cities have led to an increase in the phenomenon of “left-behind” elderly individuals. This forced social isolation is particularly evident among elderly women who have lost their spouses (Niu et al., 2020). Chronic illnesses and physical disabilities, which are more prevalent in older age, further contribute to the risk of suicidal behavior (Wang et al., 2022). Although pharmacological treatments such as antidepressants and medical interventions can alleviate certain symptoms, they are often insufficient for the long-term reduction of suicidal behaviors in older adults, partly due to the reluctance of this population to adhere to medication-based interventions (Okolie et al., 2017).

Against this backdrop, physical activity has attracted increasing attention as a low-cost, non-pharmacological intervention. Evidence suggests that regular physical activity among older adults can improve mood, reduce depression, and enhance overall well-being (Li et al., 2024). Group-based or community-based exercise programs have been shown to foster a sense of belonging and social support, thereby contributing to enhanced mental vitality and increased life expectancy. Consequently, physical activity—especially team and group exercises—has been incorporated into international guidelines as a non-pharmacological strategy for improving mental health outcomes and extending life expectancy (Aung et al., 2021). Furthermore, while the latest systematic reviews have indicated that physical activity can serve as an effective intervention to reduce suicidal ideation and self-harming behaviors (Sadek et al., 2024), attracting older adults to participate in exercise programs remains challenging due to factors such as diminished motivation and apathy (Collado-Mateo et al., 2021). Previous studies have often highlighted the benefits of physical activity in reducing suicidal thoughts but have inadequately addressed how to effectively engage elderly individuals—particularly elderly women—in consistent physical exercise programs. There remains a significant gap in the development of structured protocols aimed at increasing participation among this sensitive and growing demographic group. Therefore, it is essential to explore innovative and diverse strategies to engage elderly women in regular physical activities and appropriately address this research gap. Previous research has reported a direct relationship between participation in group and team-based activities and increased life expectancy and mental vitality, particularly among elderly women compared to men (Stødle et al., 2019). Due to their greater emotional dependency and need for social interaction and conformity, women benefit more from team-based activities, making such interventions particularly effective in enhancing their hope for life (Doughabadi et al., 2016).

The emergence of new technologies, particularly VR, has created more attractive and safer environments that offer unique and immersive experiences in education and public health (Yondjo and Siette, 2024). VR provides a safe and enjoyable space that allows individuals to engage in physical activities without the limitations of the real world, such as fear of falling or lack of companions (Wang et al., 2025). Essentially, VR is a computer-simulated environment that enables users to interact using multiple senses, such as vision, hearing, and touch, creating a sense of presence similar to the real world (Dong et al., 2025). The VR experience typically involves the use of head-mounted displays, which distinguish it from conventional gaming by offering an immersive and unique experience (Hasselberg, 2020). Building on the immersive capabilities of VR, gamification can be integrated to further enhance user engagement and motivation in non-game settings. Gamification, defined as the deliberate use of game design elements—including point systems, progressive levels, leaderboards, rewards, and instant feedback—within non-game settings, has been shown to enhance motivation and engagement (Yu et al., 2025). By incorporating psychological, social, and technical elements, gamified interventions can maximize engagement, social connection, and behavioral adherence. Integrating VR with gamification can further enhance enjoyment and engagement, creating an innovative environment for elderly women to participate in physical activities.

In this context, the aim of the present study is to integrate general and team-based physical activities with gamification elements within an innovative and engaging VR environment to encourage elderly women to participate in exercise programs. By combining team-based activities with gamification features such as excitement, rewards, competition, and enjoyment, which have been shown to enhance motivation and engagement in educational and physical activities (Faridniya et al., 2024), the intervention seeks to motivate elderly women to engage in physical exercise.

## 2 Methods

### 2.1 Research design

This study employed an experimental research design in the form of a RCT with non-probability convenience sampling. Given the practical constraints of accessing this vulnerable population, participants were recruited using a convenience sampling strategy from eligible residents in senior care facilities. However, to enhance internal validity and control for selection bias, participants who met the inclusion criteria were randomly allocated into two groups—experimental and control—using a computer-generated randomization list prepared by a researcher not involved in the intervention delivery.

### 2.2 Participants and recruitment

The study sample included elderly women experiencing suicidal ideation who lived in Beijing. A convenience and voluntary sampling strategy was used. Using initial inclusion conditions, 148 elderly women were identified. Participants were invited using official announcements circulated at senior centers and coordinated among resident psychiatrists. After adjusting for withdrawals predicted and for the voluntary nature of participation whereby volunteers were free to withdraw at any point, and for factors of travel distance and psychiatric considerations, 120 were deemed to meet the conditions of eligibility and were then randomly allocated to two equal groups: an experimental (*n* = 60) and a control (*n* = 60) group. Participants’ ages were distributed between: 25 aged 60–65, 59 aged 66–70, 26 aged 71–75, and 10 aged 76–80. The sample size used here is greater than the minimum requirements for quasi-experimental studies. Cohen et al. (2002) opine that at least 15 people for each group would be enough to guarantee the generalizability and reliability of their findings. By using 120 people, the study not only complies but also goes beyond these standards to enhance the credibility and strength of the findings. Suicidal ideation was confirmed by board-certified psychiatrists working at the participating senior care facilities through comprehensive clinical evaluations. These evaluations adhered to the diagnostic guidelines outlined in the ICD-10 criteria for suicidal ideation (World Health Organization, 1992), encompassing assessment of persistent thoughts of self-harm, intent, and prior behaviors. The psychiatrists reviewed each participant’s complete medical record, including documented episodes of suicide attempts or preparatory behaviors recorded in the past 2 years. In addition, in-depth clinical interviews were conducted to explore the frequency, intensity, and situational triggers of suicidal thoughts, as well as protective and risk factors. This multi-source confirmation approach—combining current clinical assessment with verified historical medical records—was consistent with the standard clinical practices for suicide risk evaluation in China and ensured high diagnostic validity for participant inclusion. The inclusion factors involved the widowhood of a spouse, feelings of being abandoned, social withdrawal tendency, and high depression assessment scores.

### 2.3 Data collection tools

To measure the dependent variables, the Subjective Vitality Scale developed by Ryan and Frederick (1997) and the Hope Scale based on the model proposed by Snyder (2002) were utilized. Furthermore, improved versions of these instruments have been employed in recent studies to enhance psychometric sensitivity and applicability for older populations, as evidenced by the works of Li et al. (2024) for the Subjective Vitality Scale and Dong et al. (2025) for the Hope Scale (Li et al., 2024; Dong et al., 2025). For this study, the 19-item versions of the Subjective Vitality Scale and the Hope Scale were carefully adapted to match the cognitive and psychological characteristics of older participants. The items were reviewed and refined by a panel of clinical psychologists and psychiatrists specializing in elderly care. A pilot test with a small sample of participants from the same population confirmed that all items were understandable, culturally appropriate, and feasible for use in the target population. Construct validity and factor structure were assessed based on expert evaluation and previous research on the original scales, ensuring the adapted instruments were psychometrically sound. Although suicidal ideation was not measured directly using standardized scales such as the Beck Scale for Suicide Ideation (BSSI) or PHQ-9 Item 9, this study focused on subjective vitality and hopefulness as indirect yet validated indicators of suicide risk. Snyder emphasized that low levels of hope are strongly associated with increased suicidal thoughts and behaviors (Snyder, 2002), while Ryan and Frederick highlighted that diminished subjective vitality reflects reduced psychological well-being and higher vulnerability to self-harm (Ryan and Frederick, 1997). More recent research by Faridniya et al. (2024) supports the predictive value of these constructs for suicide risk among older adults (Dong et al., 2025). Additionally, Heisel et al. (2016), have demonstrated that enhancing hope and mental vitality can significantly reduce psychological distress and the likelihood of self-harm (Heisel et al., 2016). Based on this evidence, improvements in subjective vitality and hopefulness were considered reasonable proxies for reduced suicidal ideation, allowing for ethically safe and practically feasible assessment in this vulnerable elderly population.

Internal consistency of the tools was also established using Cronbach’s alpha and the results were found to be 0.82 for the vitality scale and 0.86 for the hope scale, denoting adequate reliability. Furthermore, to observe potential VR-associated side effects, a VR Sickness Questionnaire (VRSQ) was presented to all experimental sessions. Mild symptoms of the order of slight dizziness or nausea were experienced in some 8% of the sessions, all of which resolved spontaneously within 10 min without the need for pharmacological interference. No subjects stopped the intervention because of such symptoms, and all experimental subjects finished the protocol of the treatment in its entirety.

### 2.4 Data analysis procedure

Kolmogorov–Smirnov testing was used to analyze the normality of variable distribution, and the homogeneity of variance across groups was assessed using Levene’s test. Analysis of Covariance (ANCOVA), to analyze the research hypotheses and account for any pre-test differences, was used. The statistical analyses were done using IBM SPSS Statistics software version 26.

### 2.5 Implementation process of the training protocol for experimental and control groups

To design a safe, motivational, and targeted intervention for elderly women experiencing suicidal ideation, an interdisciplinary research team—including experts in sports science, geriatric health psychology, and VR technology—collaboratively developed an innovative training protocol. Prior to the intervention, several orientation and sensitization sessions were conducted with both participants’ families and mental health professionals to foster a supportive psychological climate conducive to voluntary and sustained participation. A clinical psychologist guided the administration of the pre-test measures and ensured that participants fully understood how to interact with the psychometric tools. Written informed consent was obtained from all participants and their legal guardians (typically their adult children), and the purpose and procedures of the study were clearly explained to ensure transparency. Importantly, no fees were charged for the educational sessions or psychological consultations. As a token of appreciation, participants received small gifts such as sportswear upon completing the program. Families were allowed to accompany participants during all sessions and were encouraged to provide motivational support throughout the intervention.

The experimental intervention was implemented using the Pico 4 Enterprise VR headset—an advanced, standalone device developed specifically for professional use in health, rehabilitation, and education. With its 4 K + resolution, wide field of view, ergonomic design, and ability to manage simultaneous group sessions, it proved ideal for delivering psychomotor interventions to elderly users. Over a five-week period, participants in the experimental group attended 15 sessions (three times per week), each lasting between 40 to 60 min. Activities included virtual group cycling, friendly boxing competitions, balance-based team games, and interactive challenges with other participants. The inclusion of virtual boxing was based on consultations with geriatric psychiatrists, who emphasized the psychological importance of incorporating competitive and high-intensity exercises at this stage of life to promote emotional resilience and survival instinct. Each session featured gamification elements such as a ranking system, scoring mechanisms, and personalized incentives (e.g., visual rewards, nostalgic video clips, and public recognition of progress in front of family members). These components were designed to boost participants’ confidence and reinforce a sense of importance and achievement. The simultaneous presence of participants within an interactive VR space not only increased motivation to continue the program but also indirectly mitigated feelings of isolation, loneliness, and worthlessness—known psychological risk factors among elderly individuals with suicidal ideation.

Participants in the control group engaged in a structured, non-interactive physical activity program carefully designed to match the experimental group in overall session duration, frequency, and general physical exertion, while deliberately excluding VR or gamification elements. Over a five-week period, participants attended three weekly sessions, each lasting 40–60 min, held in a quiet multipurpose hall at the senior care facilities under the guidance of a certified exercise instructor. Each session began with a ten-minute warm-up consisting of gentle stretching of major muscle groups and slow joint rotations, followed by 20–25 min of low-impact aerobic exercises, including seated marching, arm raises, light stepping, and slow rhythmic walking at a light effort level corresponding to 9–11 on the Borg Rating of Perceived Exertion (RPE) scale. This was complemented by 10–15 min of flexibility and balance training, such as heel-to-toe walking, single-leg stance with chair support, and torso rotations, and concluded with a five- to ten-minute cool-down featuring slow breathing and relaxation techniques. This regimen reflects widely recommended low-impact exercise protocols for elderly populations (Riebe et al., 2018) and serves as an ecologically valid, safe, and appropriate benchmark for non-technological interventions in geriatric mental health, providing a solid comparator for evaluating the added benefits of VR-based gamified exercise. The choice of this control group allows differentiation between the effects of gamified VR-based exercise and the general benefits of group-based physical activity. However, it should be noted that the study design does not allow complete disentanglement of the group-based setting versus the VR gamification component. Therefore, observed improvements may reflect contributions from both the immersive gamified experience and the social interaction inherent in group exercise. Future studies could include additional control conditions, such as individual VR exercise or group exercise without gamification, to clarify the relative contributions of these components.

## 3 Result

### 3.1 Descriptive indices of research variables

In the current research, to present more inclusive data, the descriptive statistics, i.e., the research variable’s mean and standard deviation, were given separately in the experimental and control groups. The indices thereof appear in Table 1.

**Table 1:** Descriptive statistics.

Variable	Group	Test	N	Mean	Std. deviation
Hopefulness	Control	Pre-test	60	26.35	3.90
	Control	Post-test	60	26.88	3.95
	Experiment	Pre-test	60	26.47	4.00
	Experiment	Post-test	60	33.55	4.25
Mental vitality	Control	Pre-test	60	28.20	4.00
	Control	Post-test	60	28.75	4.05
	Experiment	Pre-test	60	28.35	4.05
	Experiment	Post-test	60	32.30	4.30

### 3.2 Kolmogorov–Smirnov test for normal distribution of data

As seen in Table 2, the outcomes of the Kolmogorov–Smirnov test showed that all research variables, such as Hopefulness and Mental Vitality, showed no significant departure from normality. More specifically, in all of the pre-test and post-test scores of the control and experimental groups, the *p*-values were above 0.05. Thus, the normality assumption was adequately fulfilled to enable the application of parametric statistical procedures to subsequent hypothesis tests.

**Table 2:** Kolmogorov–Smirnov result.

Variable	Group	Test	Test statistic	Sig.
Hopefulness	Control	Pre-test	0.083	0.091
	Control	Post-test	0.074	0.200
	Experiment	Pre-test	0.088	0.057
	Experiment	Post-test	0.071	0.200
Mental vitality	Control	Pre-test	0.079	0.121
	Control	Post-test	0.075	0.190
	Experiment	Pre-test	0.083	0.088
	Experiment	Post-test	0.074	0.200

### 3.3 Levene’s test for homogeneity of variance

As shown in Table 3, the assumption of homogeneity of variance is met for all research variables (*p* > 0.05). Based on this set of assumptions, it is evident that the data from this study can be subjected to analysis of covariance (ANCOVA) to examine the differences between the two groups in terms of the dependent variables.

**Table 3:** The results of Levine’s test.

Variable	F	df1	df2	Sig.
Hopefulness	0.683	1	118	0.411
Mental Vitality	0.537	1	118	0.465

### 3.4 Research hypotheses and data analysis results

The analysis of covariance (ANCOVA) results, presented in Table 4, indicated that there were significant post-test differences between groups after controlling for pre-test scores. Particularly for Hopefulness, there was a major main effect for membership in the groups (*F*(1, 117) = 47.995, *p* < 0.001), and partial eta-squared = 0.291, meaning that 29.1% of the variance between post-test hopefulness scores was due to the treatment. Elderly women who were in the experimental condition significantly increased their hopefulness after participating in the group-based physical activity program that was combined with gamification methods within a VR context.

**Table 4:** The results of covariance analysis.

Source	Type III sum of squares	df	Mean square	F	Sig.	Partial eta squared
Hopefulness						
Pre_ hopefulness	73.227	1	73.227	31.302	0.000	0.211
Group	112.263	1	112.263	47.995	0.000	0.291
Error	273.706	117	2.339			
Mental vitality						
Pre_ mental Vitality	65.114	1	65.114	28.714	0.000	0.197
Group	90.222	1	90.222	32.166	0.000	0.215
Error	327.972	117	2.802			

Equally so, for Mental Vitality, the between-group effect was significant (F(1, 117) = 32.166, *p* < 0.001), and the partial eta-squared was 0.215, indicating that 21.5% of the variance for mental vitality outcomes was accounted for by the intervention. The intervention significantly increased levels of mental vitality among older women who were contemplating suicide. In general, these findings supply sound evidence for the effectiveness of group-based gamified exercise interventions provided by VR to substantially enhance psychological well-being outcomes for older women vulnerable to suicide.

**Figure 1:**
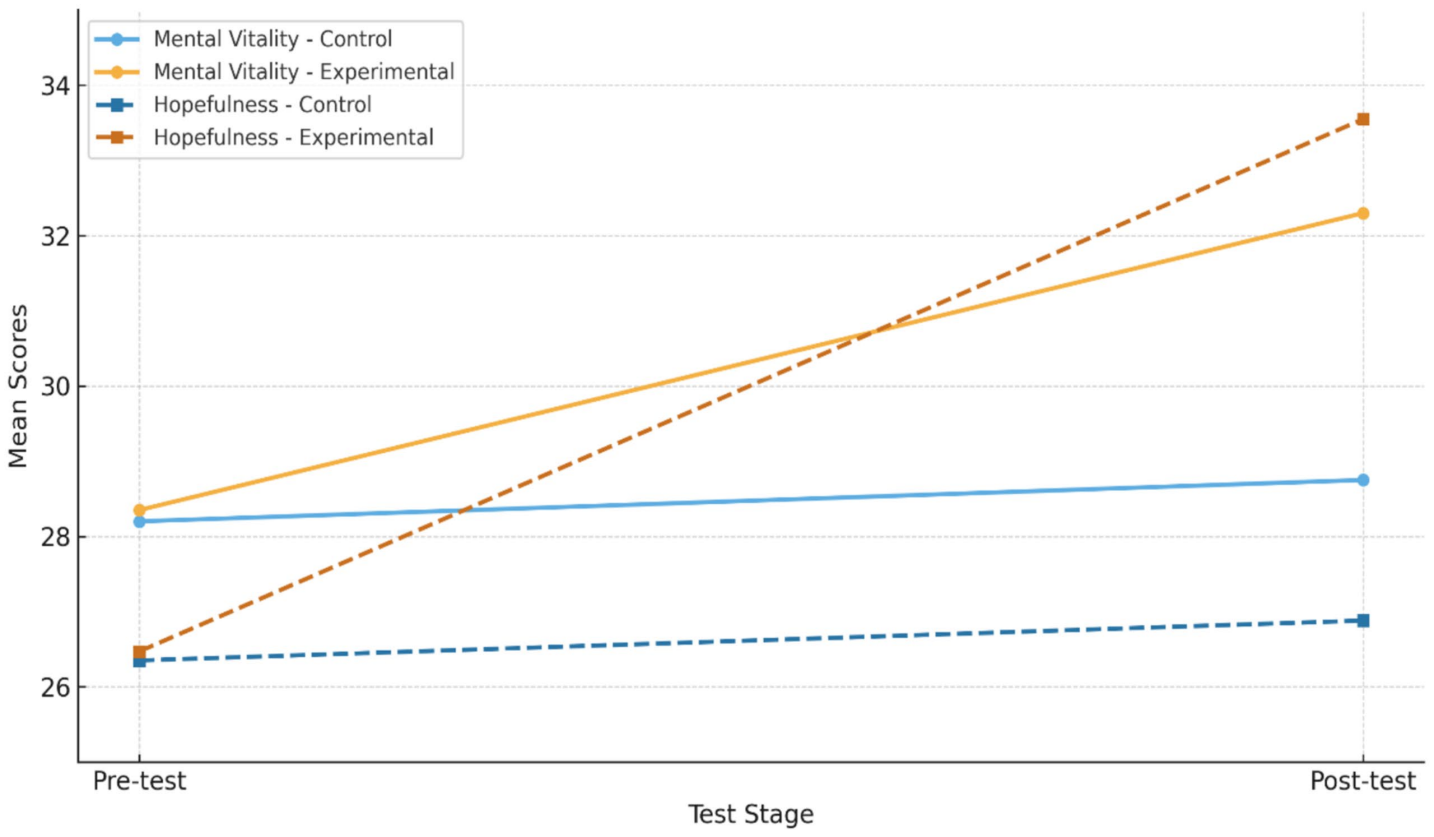
Comparison of pre-test and post-test scores.

For a more comprehensive comparison of the obtained results, the pre-test and post-test scores of both the experimental and control groups are illustrated in Figure 1.

## 4 Discussion

Studies have shown that group-based exercise can lead to significant improvements in both quality of life and mental well-being (Li et al., 2024). In response, the present study sought to design an innovative and engaging intervention tailored to this sensitive and important demographic. By incorporating VR technologies and the motivational elements of gamified group exercise, the intervention aimed to enhance mental vitality, increase life satisfaction, and ultimately reduce suicidal ideation among elderly women. The findings of this study revealed that gamified group exercise in a VR environment significantly improved life expectancy and reduced feelings of hopelessness among Chinese elderly women experiencing suicidal thoughts. These results are consistent with previous research confirming the effectiveness of VR interventions in alleviating symptoms of depression, anxiety, and phobias across various populations (Freeman et al., 2023; Hatta et al., 2022). For instance, VR applications have shown greater efficacy than traditional treatments in managing chronic pain and treating specific phobias (Dong et al., 2025; Mishkind et al., 2017). Similarly, Yondjo and Siette (2024) demonstrated that VR-based therapies can be as effective as traditional in-person interventions in reducing fear, anxiety, and avoidance behaviors.

In recent years, the use of VR as a non-pharmacological intervention for treating psychological disorders such as depression, psychosis, and phobias has received increasing attention. Numerous studies have demonstrated the effectiveness of this technology in alleviating psychological symptoms and reducing chronic pain. For example, in a randomized study involving burn patients aged 5 to 18, the use of VR games during wound dressing significantly reduced self-reported pain scores (Jain et al., 2024). Additionally, in a clinical trial, Shi et al. (2024) reported that combining cognitive-behavioral therapy (CBT) with VR and calming elements—such as a Japanese garden—led to a significant reduction in depression and anxiety scores among elderly women (Shi et al., 2024). Similarly, a systematic review by Vasodi et al. (2023) found that VR-based exercise interventions improved the overall quality of life in older adults across psychological, social, and physical dimensions, and also reduced symptoms of depression (Vasodi et al., 2023). Moreover, some studies suggest that physical activity, through improvements in physical condition and brain functioning, can increase “hope for life” and reduce suicidal tendencies. For instance, Dong et al. (2025) demonstrated that a gamified group exercise intervention delivered through VR led to a significant increase in the “life expectancy” index among older adults with suicidal ideation, by enhancing their motivation and adherence to physical activity (Li et al., 2024). These findings align with the results of the present study regarding the positive effects of VR-based exercise in enhancing mental vitality and increasing hope for life.

Group-based physical activity offers multiple psychological and physiological benefits across most types of interventions. In addition to enhancing physical capabilities such as strength, balance, and flexibility, group exercise fosters opportunities for interaction and social support, which can reduce feelings of loneliness and strengthen a sense of belonging (Stødle et al., 2019). In studies conducted on older adults, Komatsu et al. (2017) reported that participation in group exercise programs improved both physical performance and mental balance. Moreover, participants experienced stronger social connectedness and mutual support (Komatsu et al., 2017). These advantages are also maintained in virtual reality settings. For instance, an experimental study demonstrated that a group-based VR intervention significantly reduced depressive symptoms among participants, whereas the control group showed no meaningful change (Baker, 2019). Overall, VR group exercise has the potential to simultaneously improve both physical and psychological dimensions while providing an interactive and supportive environment for the elderly. In general, group activities offer greater psychological and social benefits than individual ones. Within group settings, individuals experience a sense of social support and belonging, which serves as a strong motivator for continued participation (Yen and Chiu, 2021). VR group activities can simulate peer interaction and friendly competition—elements typically absent in solitary activities—which increases older women’s motivation to remain physically active. Findings from several studies have shown that gamified VR group exercises significantly enhance self-efficacy and confidence in exercise among older adults (Jeon and Kim, 2020), whereas the absence of such social interaction in individual programs may weaken motivation. These findings align with the present study’s emphasis on utilizing group exercise to enhance a sense of coexistence and social interaction as a valuable complement to physical activity. The results underscore the importance of leveraging the potential of collective and group-based training to foster social engagement—an approach adopted in this study.

While the findings of the current research highlight the effectiveness of group-based exercise in VR environments in improving life expectancy and reducing suicidal ideation among elderly women, some studies have reported contrasting results. For example, Song et al. (2025) found that VR-based exercise interventions in older adults led to cognitive and mood improvements only in the short term, with effects diminishing after a few months (Song et al., 2025). The researchers attributed this to reduced motivation following the end of group sessions and the lack of structured follow-up. In contrast, the present study was able to sustain positive outcomes throughout the intervention, thanks to its engaging and continuous group-based game design and the active presence of motivational coaches. In another study, Jonsdottir et al. (2021) observed that older men responded more positively to VR training than women, particularly in terms of motivational and participatory indices (Jonsdottir et al., 2021). This difference may stem from gender-specific communication styles or women’s more conservative attitudes toward new technologies. However, in our study, by emphasizing interactive group activities, initial resistance among older women was largely mitigated, resulting in increased participation motivation. On the other hand, Mouatt et al. (2020) reported that although VR exercises can improve mental health, the lack of physical social contact (e.g., human touch, face-to-face interaction) may limit their effectiveness (Mouatt et al., 2020). The discrepancy may be attributed to the type of equipment used in that particular study, which relied on older versions of VR technology that lacked realism and quality. In contrast, advancements in technology have since enabled the production of high-quality headsets offering a much more immersive and realistic experience.

In this study, gamification—the application of game mechanics in non-entertainment contexts—was employed as a key strategy to enhance motivation within VR interventions. Alongside the unique immersive experience of VR and the inherent potential of group-based physical activities, gamification features such as stage-based rewards for older adults, ranking and public leaderboard announcements among family members and others (which fostered a sense of recognition and value), and the distribution of incentives at each stage, created a compelling combination to engage elderly women in physical activity. This multifaceted approach stands out from previous research, which predominantly focused either solely on the effects of VR-based training or on conventional physical exercise. Thus, a major strength of the current study lies in its integration of three powerful tools—VR, group-based exercise, and gamification—resulting in meaningful impact and highlighting the role of gamified group training in VR environments as a non-pharmacological strategy for enhancing life expectancy among older women. In line with this, psychological studies have shown that the inclusion of game-like elements such as challenges, point scoring, and reward systems in activities increases user engagement, motivation, and enjoyment (Kober et al., 2020). In the field of elderly exercise, studies such as Dong et al. (2025) found that the integration of gamification into VR-based training significantly increased older adults’ motivation to participate and continue their sessions. Consequently, this group showed better psychological outcomes—including enhanced life expectancy—compared to the control group (Dong et al., 2025). Moreover, a systematic review analyzing existing empirical studies on gamified physical activity interventions presented evidence supporting the effectiveness of gamification in increasing participation in physical activity. The review also emphasized the need for high-quality experimental studies to assess the efficacy of combining gamification with wearable devices for promoting physical activity (Mazeas et al., 2022). Additionally, in a study by Kankanamge et al. (2020), it was found that a gamified student stress-resilience program led to improvements in psychological resilience across multiple domains, including social engagement and physical activity (Kankanamge et al., 2020).

It is noteworthy that the elderly participants in the present study had no prior experience with VR. However, after the first session and their initial exposure to the VR environment, they expressed interest in using this innovative technology again. Thus, the findings of this study align with our clinical observations, indicating a significant interest among older adults in adopting VR as a future method for addressing physical and psychological challenges—especially after firsthand experience. Previous studies have highlighted the unique capabilities of this immersive technology as an effective tool for improving quality of life and well-being, particularly among elderly individuals and those in need of neuro rehabilitation. As previously discussed, VR has emerged as a transformative, non-pharmacological intervention in medicine and therapy. One of the key factors drawing attention from professionals in the healthcare field is its minimal side effects. Freeman et al. (2023) identified three primary side effects associated with VR use in the treatment of psychotic disorders: concentration difficulties while using the headset, feelings of fear during VR use, and post-use concerns (Freeman et al., 2023).

In the present study, we used the Pico 4 Enterprise headset—one of the most advanced standalone VR devices designed specifically for professional applications in healthcare, rehabilitation, and education. Our goal was to maximize the comfort and ease of use for elderly participants during their VR experience. As previously mentioned, to monitor potential side effects, the VR Sickness Questionnaire (VRSQ) was administered during all experimental sessions. Approximately 8% of the sessions involved mild symptoms such as light-headedness or slight nausea, all of which resolved within 10 min without any pharmacological intervention. None of the participants discontinued the intervention due to these symptoms, and all members of the experimental group completed the entire intervention period. The results demonstrated the effectiveness of group-based, gamified VR exercise programs in improving mental vitality, satisfaction, and life expectancy while reducing suicidal ideation among older women. These findings suggest that such an approach may serve as an innovative and appealing strategy in mental health promotion and suicide prevention programs for this vulnerable population.

## 5 Conclusion

The current research presents strong evidence that gamified group physical activities administered within a VR setting can be a viable, non-drug-based therapeutic option for maximizing life expectancy and alleviating suicidal ideation among elderly Chinese women. Through increased social connectedness, sustained psychological vitality, and heightened motivation through novel technological interactions, the intervention addressed salient psychosocial risk factors for suicide risk that are common among older adult women. Results identify the distinct promise of incorporating VR technology with group physical activity and gamification mechanisms for providing benefits that are multifaceted—enhancing physical activity, as well as emotional coping skills, social belonging, and optimism about the future. Importantly, people’s hesitations at the start were attenuated through the immersive quality of VR, as well as the controlled group setting, leading to increased adherence to the program and satisfaction with the program. Considering the accelerating international spread of elderly suicide rates, especially among socially isolated women with experiences of loss, together with chronic diseases, the current research underlines the pressing need to introduce novel, technology-based interventions into international public health practices. Future research must test these results with culturally diverse populations as well as healthcare settings, lengthen follow-up periods to gather information regarding sustained effects, as well as investigate gender-based variations to optimize participation as well as benefits.

### 5.1 Managerial and practical implications

The findings of this study provide clear, actionable insights for clinical, community, and managerial stakeholders involved in elderly mental health and suicide prevention. Implementing gamified group-based VR exercise programs in senior care facilities, community centers, or outpatient settings can create a motivating, socially supportive, and cognitively stimulating environment that enhances subjective vitality and hopefulness among high-risk elderly women. Program design should emphasize interactive elements, including team-based challenges, scoring systems, stage-based rewards, and public recognition, to maintain engagement and foster a sense of achievement. Temporal patterns of suicide highlight critical windows of vulnerability, with studies showing peaks in suicide attempts during the late afternoon and early evening, particularly among high-risk elderly populations (Kanani and Sheikh, 2024). Aligning VR-based, gamified group exercise sessions with these periods could enhance preventive impact and maximize engagement. By scheduling interventions during these critical windows, clinicians and community practitioners may leverage the heightened susceptibility to optimize the benefits of mental vitality and hopefulness, thereby providing actionable guidance for real-world implementation. Moreover, training staff in VR operation, participant monitoring, and safe intervention delivery is essential to ensure ethical and effective application. These insights demonstrate the potential for integrating innovative, technology-driven interventions into geriatric mental health programs, addressing cognitive, emotional, and social dimensions simultaneously while offering scalable, cost-effective strategies for suicide prevention.

### 5.2 Limitations and future research

Several limitations should be considered when interpreting the findings of this study. First, the study was not blinded, which may introduce observer and participant expectancy biases. Both the experimental and control groups were actively supervised, potentially influencing participants’ motivation, engagement, and self-reported outcomes. While validated questionnaires and standardized protocols were used to mitigate bias, awareness of group assignment may have affected the results. Future research should implement blinding where feasible, or employ additional objective outcome measures such as physiological or behavioral indicators, to strengthen internal validity. Second, all participants were recruited from senior care facilities in Beijing, limiting the generalizability of the findings. Regional, socioeconomic, and cultural variations were not addressed, and responses to VR-based interventions may differ in other populations or geographic contexts. Future studies should recruit participants from diverse locations and cultural backgrounds to improve external validity and provide more representative insights into the applicability of gamified VR interventions. Third, approximately 8% of participants experienced mild VR-related symptoms such as dizziness or nausea. While these individuals did not differ from the rest of the sample in baseline characteristics or post-test outcomes, and no participants discontinued the intervention, temporary discomfort could have influenced engagement or subjective responses. Future research could explore adaptive VR settings, session duration adjustments, or pre-conditioning strategies to minimize such side effects in elderly populations. Fourth, this study assessed outcomes only immediately after the five-week intervention, without any extended follow-up period. As a result, the durability and long-term sustainability of the observed improvements remain uncertain. Given the importance of sustained effects in suicide-prevention interventions, future research should incorporate longitudinal follow-up assessments (e.g., at 3, 6, and 12 months) to evaluate whether the benefits of VR-based gamified group exercise are maintained over time. Also it should be noted that the study design does not allow for complete disentanglement of the effects of the group-based setting versus the VR gamification component. Therefore, the observed improvements in subjective vitality and hopefulness may reflect contributions from both the immersive gamified experience and the social interaction inherent in group exercise. Future studies could include additional control conditions (e.g., individual VR exercise or group exercise without gamification) to clarify the relative contributions of these components. Finally, although the study focused on indirect indicators of suicidal risk such as hopefulness and subjective vitality, direct measurement of suicidal ideation was not performed due to ethical constraints. Future research could consider carefully designed, ethically approved methods for direct assessment, or longitudinal designs to examine the long-term impact of VR-based interventions on actual suicide-related behaviors and cognitive-emotional resilience.

## Data Availability

The raw data supporting the conclusions of this article will be made available by the authors, without undue reservation.
